# Ultrasound-Guided Iliopsoas Tenotomy for Iliopsoas Tendon Impingement: Surgical Technique in Cadaveric Models

**DOI:** 10.1016/j.eats.2024.103254

**Published:** 2024-09-30

**Authors:** Pablo Froidefond, Rayan Fairag, Alexandre Rudel, Peter N. Chalmers, Nicolas Bronsard, Régis Bernard de Dompsure, Jean-François Gonzalez, Grégoire Micicoi

**Affiliations:** a University Institute for Locomotion and Sports (iULS), Pasteur 2 Hospital, University Côte d'Azur, Nice, France; b Laboratoire d'Anatomie Normale, Faculté de Médecine de Nice, University of Côte d'Azur, Nice, France; c Department of Radiology, Centre Hospitalier Universitaire de Nice, Hôpital Pasteur 2, Nice, France; d Department of Orthopaedic Surgery, University of Utah, Salt Lake City, Utah, U.S.A.

## Abstract

Iliopsoas tendon impingement after total hip replacement has been reported with an incidence of up to 8.3%. Iliopsoas tendon impingement has also been observed in young active patients engaged in extreme sports. In such cases, surgical iliopsoas tendon release or tenotomy may be considered to improve anterior hip pain and function. Currently, iliopsoas tenotomy is performed either in an open manner or arthroscopically. This article describes a surgical technique using percutaneous ultrasound-guided iliopsoas tenotomy in cadaveric models. We perform the release at the acetabulum because it is safe and provides good sonographic visualization. This study describes the effectiveness of percutaneous iliopsoas tendon tenotomy under ultrasound guidance. However, clinical studies are warranted to confirm these findings. This minimally invasive procedure opens opportunities for clinical applications, comparing outcomes with those of standard approaches and conducting cost analyses. It may offer a cost-effective outpatient clinic option with local anesthesia, avoiding operating room expenses.

The iliopsoas tendon (IPT), formed by the iliacus and psoas muscles, crosses the pelvic rim at the inguinal ligament level and attaches at the femur’s lesser trochanter.^1^ Its anterior position relative to the acetabular wall, separated by the iliopsoas bursa, can lead to impingement after total hip replacement (THR).^2^ Iliopsoas tendon impingement (IPTI) after THR affects up to 8.3% of cases, causing groin pain that limits hip extension.^3^, ^4^, ^5^ IPTI can also occur in young, active individuals, causing snapping hip syndrome. Surgical iliopsoas tendon tenotomy (IPTT) is an effective treatment when conservative measures fail.^6^^,^^7^ Various surgical techniques, including open, endoscopic, and arthroscopic approaches, have been proposed for IPTT in different locations.^6^^,^^8^ Although arthroscopic methods reduce complications,^9^^,^^10^ they still pose risks such as hematoma, infection, paresthesia, and muscle weakness. The surgical indication is IPTI after THR refractory to nonoperative treatment.^11^ In patients with a prominent acetabular component, psoas tenotomy can also be considered first for therapeutic purposes before opting for morbid revision surgery.^12^^,^^13^ Our study introduces the feasibility of IPTT under ultrasound (US) guidance in cadaveric models, which offers a percutaneous approach, potentially reducing complications and allowing local anesthesia.

## Surgical Technique: US-Guided IPTT

**Fig 1:**
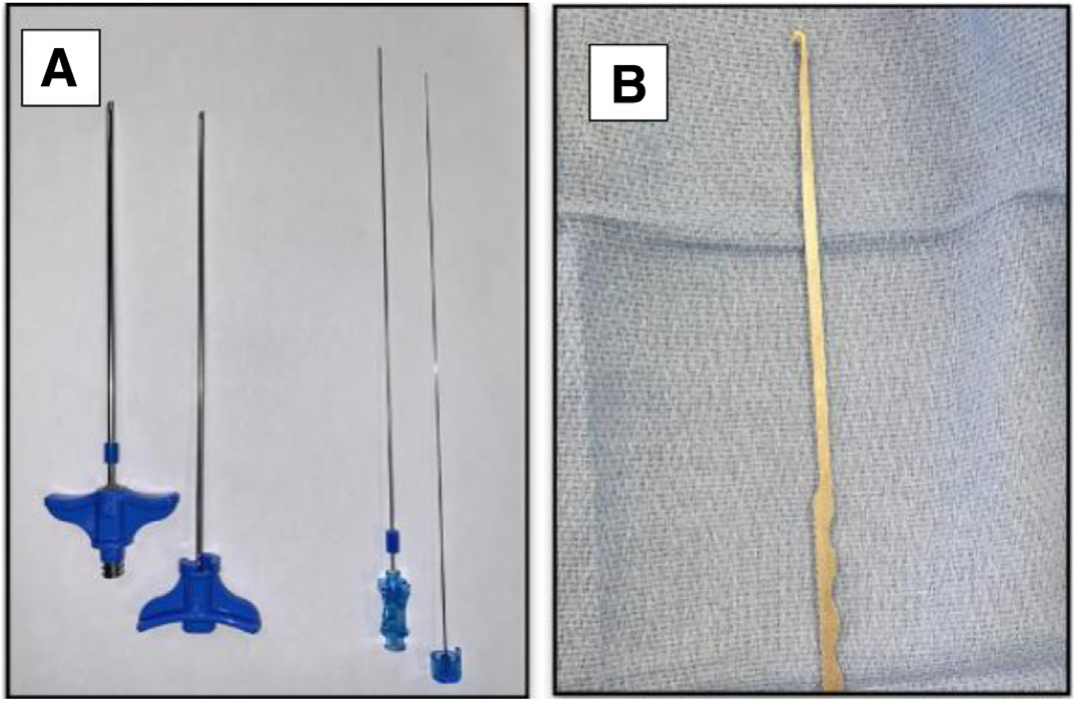
(A) T’AM kit with composed 13-gauge trocar (left) and 2 Chiba 20-gauge needles (right). (B) Acufex hooked blade.

A MyLab Alpha US machine with a linear 8- to 12-MHz transducer (ESOATE Medical SAS, Saint-Germain-en-Laye, France) is used (Video 1). US-guided IPTT is performed using a 3-mm hooked blade (Acufex; Smith & Nephew, London, England). The T’AM kit (Thiebaud, Thenon-les-bains, France), normally used for guided vertebroplasty, is used to introduce the cutting Acufex hooked blade (Fig 1). A Chiba 20-gauge needle (part of the T’AM kit) serves as a guide before introducing the trocar. The Chiba needle is one of the most commonly used percutaneous or biopsy access needles. It is a thin-walled straight needle with a beveled tip angle of 30°.

**Fig 2:**
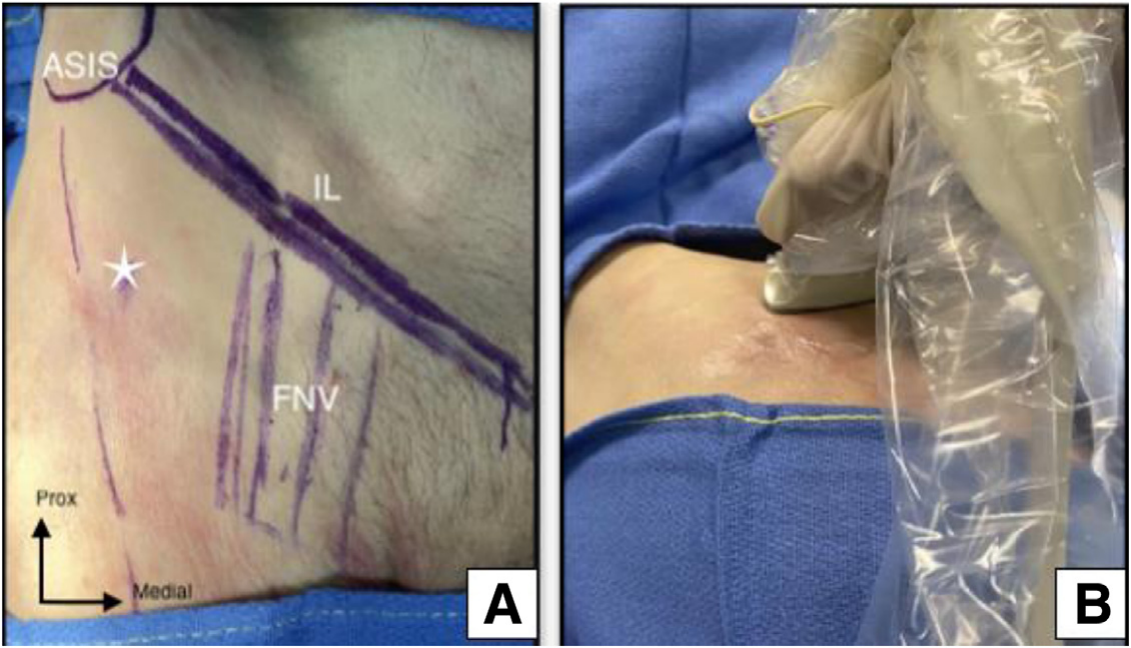
Top view of skin landmarks (A) and ultrasound transducer positioning (B) in left hip and supine cadaveric model. Care should be taken to avoid the nearby femoral neurovascular bundle (FNV). The star indicates the optimal entry point of the needle approach. (ASIS, anterior superior iliac spine; IL, inguinal ligament; Prox, proximal.)

**Fig 3:**
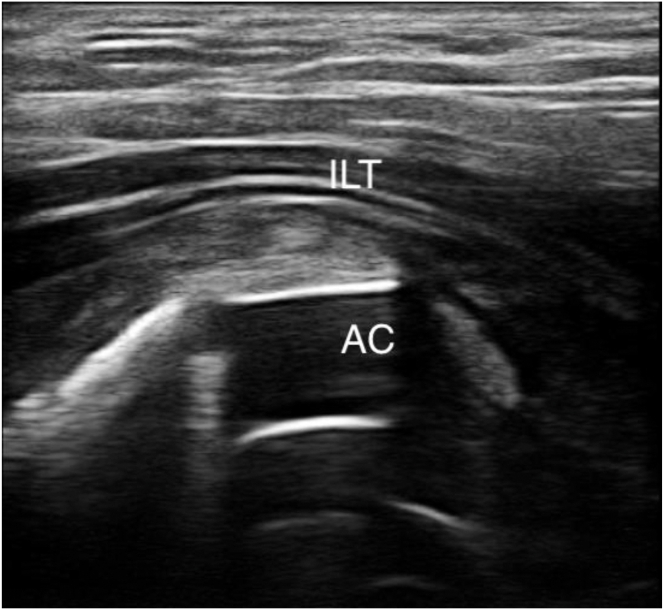
Ultrasonographic axial oblique view of iliopsoas tendon (ILT) with acetabular cup (AC) impingement.

The patient is positioned supine, and the US transducer (MyLab Alpha) is placed axially over the inguinal region to locate the iliopsoas musculotendinous unit near the anterosuperior aspect of the acetabular rim (Fig 2). Proximal and distal scans are performed to assess the iliopsoas belly muscle and its tendinous portion concerning the adjacent femoral neurovascular bundle. Subsequently, the transducer is rotated sagittally to reveal the long axis of the IPT and identify adjacent structures, particularly the lateral femoral cutaneous nerve, marked to prevent injury during needle insertion (Fig 3).

**Fig 4:**
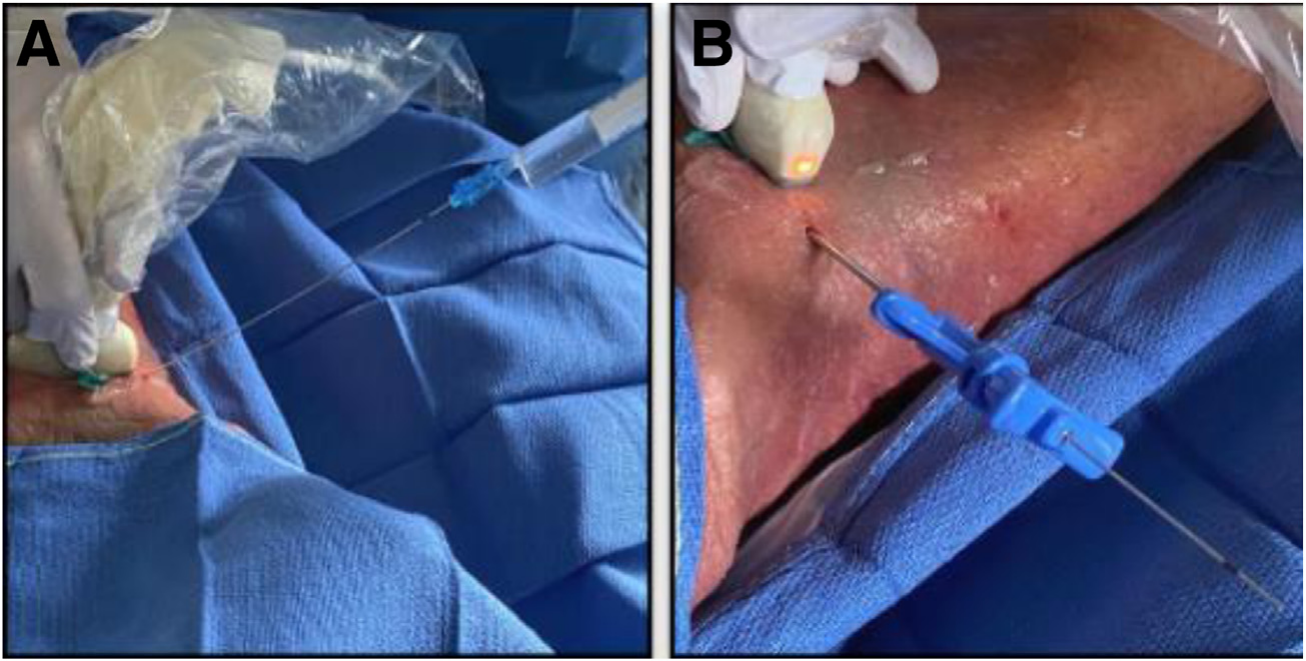
(A) Top view of Chiba 20-gauge needle entry point under ultrasound guidance with 10-mL syringe filled with water to enhance visualization through hydrodissection (right hip in supine cadaveric model). (B) Side view of 13-gauge trocar (T’AM kit) introduced over needle to create passage for hooked blade.

Under US guidance, a Chiba 20-gauge needle (T’AM kit) is inserted to aim at the level where the IPT crosses the acetabular rim near the iliopectineal eminence (Fig 3). Special care is taken to avoid the nearby neurovascular bundle during needle introduction. To enhance visualization and displace adjacent structures from the needle tip, injection of a 10-mL syringe filled with water is performed, and a 3-mm skin incision is made to allow the tenotomy device to advance through it. A 13-gauge trocar is introduced over the needle to create a passage for the hooked blade (Fig 4).

**Fig 5:**
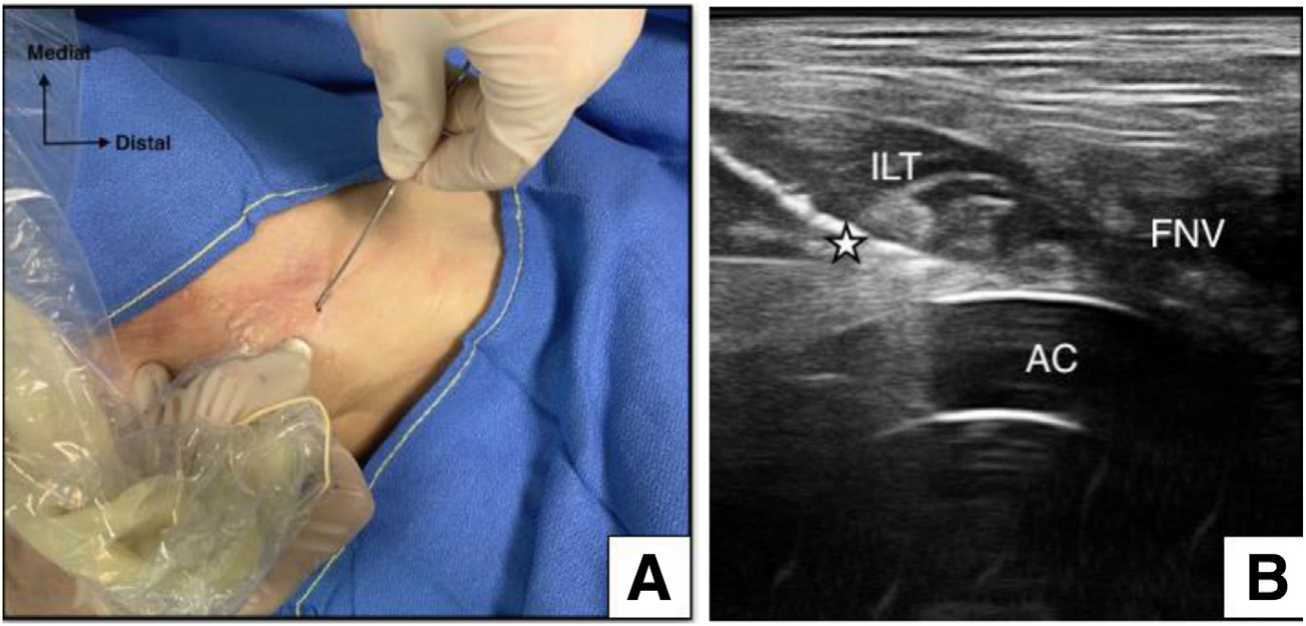
(A) Top view of hooked blade introduction guided from lateral to medial to iliopsoas tendon under ultrasound-guided control with positioning orthogonal to its fibers lying over acetabular cup. (B) Right hip in supine cadaveric model. The star indicates tenotomy of the fibers. (AC, acetabular cup; FNV, femoral neurovascular bundle; ILT, iliopsoas tendon.)

After the removal of the 13-gauge trocar, the hooked blade is positioned adjacent to the IPT and oriented perpendicular to its fibers. The blade is introduced with the cutting tip rotated 90° to minimize damage to surrounding tissues. Once the hook is placed over the IPT, it is turned orthogonal to perform the tenotomy (Fig 5A). IPTT is carried out distal to the anterior inferior iliac spine at the acetabular rim. Real-time monitoring with US allows for precise control as the hooked blade cuts through the tendon fibers (Fig 5B). The tenotomy's completion is determined by US visualization, loss of resistance during blade advancement, and direct visualization after dissection.

## Discussion

US-guided psoas tenotomy offers the advantage of reducing complications (nerve paresthesia, hematoma, infection, and painful bursitis) compared with traditional methods while enabling a percutaneous procedure under local anesthesia with surgical precision using US guidance real-time monitoring. However, disadvantages include the requirement for experienced operators, the limitations of a cadaveric study, and the ongoing debate about the ideal tenotomy location.

The primary risks associated with this procedure are vascular injury to the femoral neurovascular bundle and damage to the lateral femoral cutaneous nerve. Because of these risks, an adequate learning curve is essential for operators with limited experience in ultrasonography. Pearls and pitfalls of this percutaneous surgical technique are summarized in Table 1. It is important to note that performing this tenotomy procedure can be challenging in overweight or obese patients because of increased tissue depth, which may affect US visualization and procedural ease (Table 2).

**Table 1:** Pearls and Pitfalls of US-Guided Iliopsoas Tendon Tenotomy in Cadaveric Models

Pearls	Pitfalls
The ASIS should be identified.	Insertion should not be performed proximal to the ASIS and the inguinal ligament.
Proximal-to-distal axial-plane US scans over the inguinal region should be obtained to identify the iliopsoas musculotendinous unit. The FNVB and LFCN are located in the axial and sagittal planes.	Any anatomic variance of the iliopsoas tendon (1 bundle, 2 bundles, or 3 bundles) should be noted.
The optimal entry point is lateral to medial to avoid the LFCN.	The cutting device should not be inserted too vertically to allow for lateral-to-medial maneuvering room over the iliopsoas tendon.
The surgeon should repeatedly advance and retract slowly in a sawing action through the tendon fibers to complete the tenotomy under US control.	Injury to the LFCN, femoral nerve, femoral artery, or femoral vein is possible. Anterior hip capsule injury is possible. Injury to the surrounding soft tissues is possible.
Once the iliopsoas tenotomy is completed, the surgeon should remove the hook with the cutting blade facing downward to avoid injury to soft tissues.	There is a possibility of incomplete tenotomy related to lack of muscle tension or difficult tactile feedback of the tenotomy.

ASIS, anterior superior iliac spine; FNVB, femoral neurovascular bundle; LFCN, lateral femoral cutaneous nerve; US, ultrasound.

**Table 2:** Advantages and Disadvantages of US-Guided Iliopsoas Tendon Tenotomy in Cadaveric Models

Advantages
Percutaneous approach
Control of NVB under US guidance
Good iliopsoas tendon sonographic visualization at acetabular rim
No surgical procedure (operating room, patient positioning, image intensifier, and general anesthesia)
Potential outpatient clinic setting procedure
Disadvantages
Experienced sonographer required
Steep training curve
Cadaveric models lack muscle tension
Contraindications for high BMI

BMI, body mass index; NVB, neurovascular bundle; US, ultrasound.

This minimally invasive procedure opens opportunities for clinical applications, comparing outcomes with those of standard approaches and conducting cost analyses. It may offer a cost-effective outpatient clinic option with local anesthesia, avoiding operating room expenses.

## Disclosures

All authors (P.F., R.F., A.R., P.N.C., N.B., R.B.d.D., J-F.G., G.M.) declare that they have no known competing financial interests or personal relationships that could have appeared to influence the work reported in this paper.

## Supplementary Data

Video 1 Ultrasound-guided iliopsoas tendon tenotomy technique in supine cadaveric model. The procedure is performed on a left native hip.
